# ACCURACY OF MINIMUM DATA SET FALL ASSESSMENTS IN LONG-TERM CARE RESIDENTS

**DOI:** 10.1093/geroni/igae098.3781

**Published:** 2024-12-31

**Authors:** Laura Graham, Yongmei Li, Chintan Dave, Michelle Odden

**Affiliations:** VA HERC, VA Palo Alto Health Care System, VA Palo Alto Health Care System, California, United States; Stanford, Palo Alto, California, United States; Rutgers University, New Brunswick, New Jersey, United States; Stanford University, Palo Alto, California, United States

## Abstract

The Minimum Data Set (MDS) is commonly used to ascertain falls among long-term care residents for research and quality improvement. However, studies have shown that MDS may underreport falls compared to chart abstraction. Natural Language Processing (NLP) algorithms to detect falls are limited but have demonstrated effectiveness in identifying falls in medical records. Thus, we aimed to develop and validate a rule-based NLP algorithm to identify falls in Veterans Health Administration (VA) long-term care residents and compare it to MDS. To do so, we identified a national cohort of patients receiving care at 114 VA long-term care facilities (10/1/2006-9/30/2019). Using text mining in VA clinic notes, we developed a rule-based NLP algorithm to identify falls. The algorithm was validated using manual chart abstraction on 200 patients (positive predictive value [PPV] 0.99). We then compared the results of the NLP algorithm to the MDS assessment, which identifies a fall within the past 30 days at the time of the assessment, using sensitivity, specificity, and PPV. In our cohort of 45,183 patients, we identified 154,165 falls (7.8 per 1,000 days) among 22,331 residents using NLP compared to 16,356 falls (0.8 per 1,000 days) among 8,859 residents using MDS. Compared to NLP, the MDS had a sensitivity of 0.30, a specificity of 0.91, and a PPV of 0.77. Our findings suggest that MDS may dramatically undercount falls among long-term care residents, potentially leading to misclassification in research studies. Future studies should focus on alternative methods of fall detection in retrospective cohorts.

